# Variability of Major Phenyletanes and Phenylpropanoids in 16-Year-Old *Rhodiola rosea* L. Clones in Norway

**DOI:** 10.3390/molecules25153463

**Published:** 2020-07-30

**Authors:** Abdelhameed Elameen, Vera M. Kosman, Mette Thomsen, Olga N. Pozharitskaya, Alexander N. Shikov

**Affiliations:** 1 NIBIO, Norwegian Institute for Bioeconomy Research, Høghskoleveien 7, N-1431 Ås, Norway; 2 St. Petersburg Institute of Pharmacy, Leningrad Region, Vsevolozhsky District, P 245 188663 Kuzmolovo, Russia; kosmanvm@mail.ru; 3 NIBIO, Norwegian Institute for Bioeconomy Research, Øst Apelsvoll, 2849 Kapp, Norway; mette.thomsen@nibio.no; 4 Murmansk Marine Biological Institute of the Russian Academy of Sciences (MMBI RAS), Vladimirskaya, 17, 183010 Murmansk, Russia; olgapozhar@mail.ru; 5 St. Petersburg State Chemical Pharmaceutical University, Prof. Popov, 14, 197376 Saint-Petersburg, Russia; spb.pharmacy@gmail.com

**Keywords:** bioactive compounds, cinnamyl alcohol, freeze-drying, HPLC, *Rhodiola rosea*, rosavin, rosin, salidroside, tyrosol

## Abstract

*Rhodiola rosea* L. (roseroot) is an adaptogen plant belonging to the Crassulaceae family. The broad spectrum of biological activity of *R. rosea* is attributed to its major phenyletanes and phenylpropanoids: rosavin, salidroside, rosin, cinnamyl alcohol, and tyrosol. In this study, we compared the content of phenyletanes and phenylpropanoids in rhizomes of *R. rosea* from the Norwegian germplasm collection collected in 2004 and in 2017. In general, the content of these bioactive compounds in 2017 was significantly higher than that observed in 2004. The freeze-drying method increased the concentration of all phenyletanes and phenylpropanoids in rhizomes compared with conventional drying at 70 °C. As far as we know, the content of salidroside (51.0 mg g^−1^) observed in this study is the highest ever detected in *Rhodiola* spp. Long-term vegetative propagation and high genetic diversity of *R. rosea* together with the freeze-drying method may have led to the high content of the bioactive compounds observed in the current study.

## 1. Introduction

*Rhodiola rosea* L. (accepted name *Sedum roseum* (L.) Scop. according to www.theplantlist.org), also known as roseroot, artic-root, or golden root, is an adaptogen plant belonging to the Crassulaceae family [1,2,3,4,5]. *R. rosea* is distributed in China, Russia, Central, Northern Europe, and North America. In Scandinavia, *R. rosea* has been used as a traditional adaptogen agent for a long time, and the Vikings used it to enhance their physical performance, strength, and endurance [6]. Currently, many studies claim that *R. rosea* extracts and its bioactive compounds have antioxidant and antibacterial effects and therapeutic applications including for brain diseases and cancer therapy [3,7,8,9,10,11,12,13].

*R. rosea* rhizomes are mostly collected from natural habitats, and due to the intensive collection, natural populations are highly threatened [14,15]. *R. rosea* has become a threatened plant species in many countries [15,16,17,18], and in Bulgaria the species is listed on the red list as an endangered plant species [19]. The intensive harvesting of the natural populations as a consequence of the rapidly growing demand and the high price of the raw material resulted in increased pressure on the natural *R. rosea* habitats [15,20]. Therefore, the introduction and cultivation of *R. rosea* is an important issue to preserve and to maintain the genetic diversity of the species. Norway is one of the countries in Europe where *R. rosea* is most abundant and distributed all over the country and is known to have high genetic diversity [21,22]. The Norwegian germplasm collection of *R. rosea* was established in 2001 and originated from different counties, was vegetatively propagated, and was grown under uniform environmental conditions for 19 growing seasons [21].

Among more than 150 bioactive compounds identified in *R. rosea* [3], the major compounds with important pharmacological values are salidroside, tyrosol, rosavin, rosin, and cinnamyl alcohol, which belong to phenyletanes and phenylpropanoids [7,13,23,24,25,26]. The concentration of the bioactive compounds in *R. rosea* meeting the pharmacopeia requirement and the limited genetic resources of the species are the main challenges of meeting the demand of the global market [20,21,26,27]. Due to their bioactivity effects, salidroside and rosavin have been recommended to be used as markers for quality evaluation of *R. rosea* [3,7,10,13,28,29]. Thus, *R. rosea* extracts used in most clinical studies have been standardized to contain 0.8–1% salidroside and a minimum of 3% rosavin [30,31]. One challenging aspect is that salidroside and rosavin content in most *R. rosea* species is low [26]. Some studies were performed to increase the content of these important compounds using synthetic biology and other biotechnology methods [32,33]. The age of plants, method of drying plant material, and temperature could affect the yield of active compounds.

The objective of this study was to investigate the influence of long-term vegetative propagation on the production of the major phenyletanes and phenylpropanoids rosavin, salidroside, rosin, cinnamyl alcohol, and tyrosol by rhizomes of *R. rosea.* We also studied the impact of the freeze-drying method at −130 °C versus conventional drying at 70 °C on the content of bioactive compounds. To achieve these goals, seven clones of *R. rosea* were studied.

## 2. Results

### 2.1. Comparison of Phenyletanes and Phenylpropanoids Content in R. rosea Clones

The amount of water lost using drying at 70 °C was between 74.19% and 78.28%, while water lost using freeze-drying at −130 °C was slightly higher and varied from 75.17% to 78.62% (the difference was not statistically significant, *p* > 0.05). The amount of water lost varied among *R. rosea* clones; clone M7 lost 75% water content, while clone M6 lost up to 78% (Table 1).

**Figure 1:**
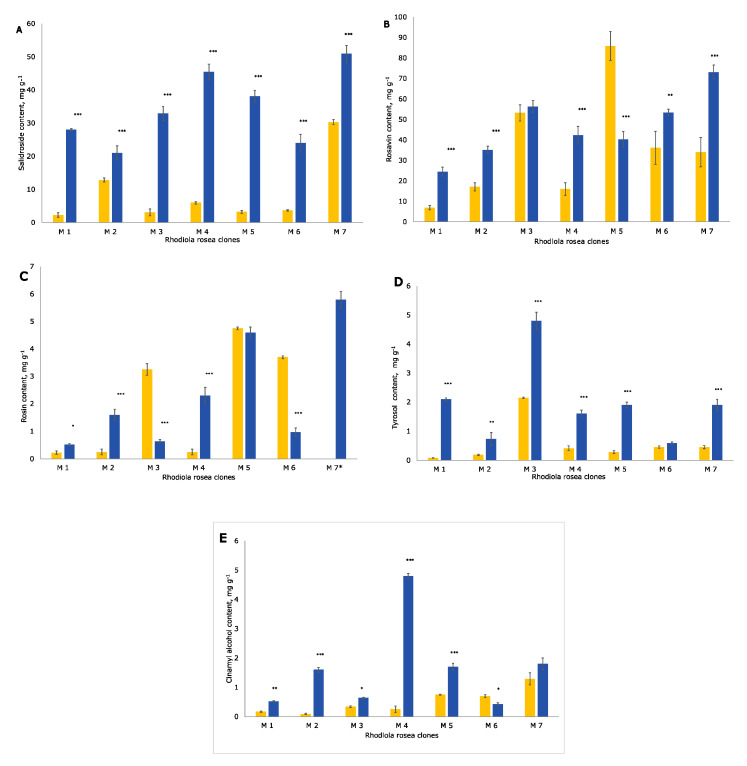
The concentration of the bioactive compounds in the seven clones (M1, M2, M3, M4, M5, M6, and M7) of *R. rosea*: (**A**) salidroside; (**B**) rosavin; (**C**) rosin; (**D**) tyrosol; and (**E**) cinnamyl alcohol; *R. rosea* analyzed in 2004 (orange color); produced in 2017 (blue color); mean concentration in mg g^−1^ ± standard deviation. Only results obtained using the freeze-drying method are presented in the figure. * Rosin was not studied in 2004 in clone M7. * *p* < 0.05, ** *p* < 0.01, and *** *p* < 0.001 for all compounds compared to the samples analyzed in 2004.

HPLC is a widely used technique for quantifying salidroside, tyrosol, rosavin, rosin, and cinnamyl alcohol in *R. rosea* [22,23,26,34,35]. HPLC analyses showed the high content and large diversity in the concentration of phenyletanes and phenylpropanoids in the *R. rosea* clones collected in 2017 (Table 1). All five bioactive compounds were detected in all seven clones in 2004 and in 2017 (Figure 1). In 2004, the highest content of salidroside and cinnamyl alcohol was found in clone M7, rosavin and rosin dominated in M5, while the maximal concentration of tyrosol was in clone M3. In 2017, the highest content of salidroside, rosavin, and rosin was observed in clone M7. Tyrosol was abundant in clone M3 as was cinnamyl alcohol in clone M4.

**Figure 2:**
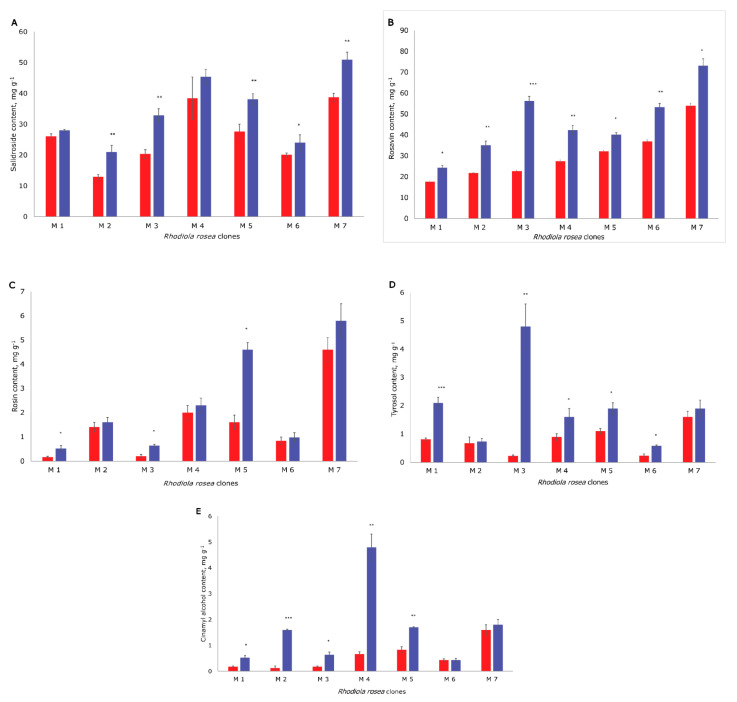
The concentration of the bioactive compounds in the seven clones (M1, M2, M3, M4, M5, M6, and M7) of *R. rosea*: (**A**) salidroside; (**B**) rosavin; (**C**) rosin; (**D**) tyrosol; and (**E**) cinnamyl alcohol; samples dried at 70 °C (red color); freeze-dried at −130 °C (blue); mean concentration in mg g^−1^ ± standard deviation. * *p* < 0.05, ** *p* < 0.01, and *** *p* < 0.001 for all compounds compared to the samples dried at 70 °C.

Significantly increased content of bioactive compounds of *R. rosea* was achieved by using freeze-drying at −130 °C rather than drying at 70 °C (Table 2; Figure 2).

### 2.2. Statistical Analyses

Pearson correlation analysis showed high significant correlations among the five compounds (rosavin, salidroside, rosin, cinnamyl alcohol, and tyrosol) measured in the *R. rosea* clones studied (Table 3). The highest significant correlation was found for the production of the rosavin and salidroside (r = 0.9025; *p* ≤ 0.001). The lowest correlation was found between salidroside and rosin (0.2648). Norwegian *R. rosea* clones contained high levels of bioactive compounds that exceeded the level required for *R. rosea* to be used for clinical treatment (Table 3).

Regression analysis showed no significant correlation between the results from a previously performed amplified fragment length polymorphism (AFLP) analysis [21] and the chemical composition of *R. rosea* clones in this study.

## 3. Discussion

In this study, we compared the content of phenyletanes and phenylpropanoids rosavin, salidroside, rosin, cinnamyl alcohol, and tyrosol in plants from the Norwegian germplasm collection of *R. rosea* first collected in 2001 and analyzed in 2004 [26] and collected and analyzed in 2017 (Figure 1). In general, the content of these bioactive compounds investigated in the current study was significantly higher than that observed in 2004. Rosavin content in clone M5 was lower. Rosin was increased in three clones (M1, M2, and M4) and was decreased in three clones (M3, M5, and M6) and was very high in clone 7, which was not studied previously [26].

Due to the bioactive effects, salidroside and rosavin have been proposed to be used as markers for quality evaluation of *R. rosea* [28,29]. The minimal concentration of salidroside and rosavin in high-quality rhizomes of *R. rosea* is recommended to be 0.8–1% and 3%, respectively [30,31,36]. The levels of these two bioactive compounds were either low or absent in many samples of Rhodiola species market products [37,38] reported in the literature (Table 4). The concentrations of bioactive compounds reported in Table 4 were calculated for absolute dry mass of rhizomes. Authors of some articles did not specify whether the concentrations of bioactive compounds were calculated for absolute dry mass of rhizomes or not. These articles are marked with ND in the table. Both chemical synthesis and biosynthesis methods were applied for the production of salidroside and rosavin [33,39,40,41,42]. However, the maximum salidroside and rosavin obtained from these studies was limited and as low as 0.569 mg mL^−1^ and 1.107 mg mL^−1^ [32,33], respectively. On the other hand, in our study, a higher content of up to 51.0 mg g^−1^ of salidroside and 73.1 mg g^−1^ of rosavin was observed. Apparently, the freeze-drying method increased the concentration of all phenyletanes and phenylpropanoids compared with conventional drying at 70 °C. As far as we know, the content of salidroside observed in this study was the highest ever detected in *Rhodiola* spp. Although salidroside was the main investigated bioactive compound in clinical studies, treatment with this compound alone was less effective than with *R. rosea* extract, suggesting a synergistic effect with other compounds [43]. The highest significant correlation detected between rosavin and salidroside in this study showed a high quality and quantity in Norwegian *R. rosea*, which is suitable to meet the global pharmaceutical demand.

The bioactive compounds’ variability in *R. rosea*, between years, was reported from two to three, four to five, four to seven, and four to nine years [14,35,46,55]. To our knowledge, this is the first study that investigated the variability of active compounds in *R. rosea* from three to twelve years. Our 16-year-old *R. rosea* rhizomes showed increased content of these bioactive compounds. These results are in line with the results of Peschel et al. (2016) [56], who have studied 9-year-old rhizomes. Our results may support the hypothesis [26] that long-term vegetative propagation increases the accumulation of these bioactive compounds in *R. rosea***.** Long-term vegetative propagation might lead to genetic adaptability and stability of these bioactive compounds in the absence of crossbreeding and seed propagation. The high content of salidroside and rosavin detected in the samples of *R. rosea* rhizomes from our germplasm collection in Norway, compared to the low quantity detected in other studies (Table 4), may be explained by the light of the longer days in spring and summer in northern latitudes that promote increased metabolite production. Furthermore, Galambosi et al. (2009) found that the content of total flavonoids of the Nordic *R. rosea* was higher than those originating from Central European countries [57].

The AFLP molecular data matrix of these seven R. rosea clones [21] was correlated with their chemical data. No significant association was found between the results from our previous AFLP analysis [21] and the chemical composition of the *R. rosea* clones in the current study. This result is in agreement with our previous study [26]. This might be because AFLP markers were limited to non-coding regions of the R. *rosea* genome. Thus, a further genomic study investigating genes responsible for the production of these bioactive compounds is required.

The various methods used to dry and extract the bioactive compounds of *R. rosea* have a large impact on the yield of phenyletanes and phenylpropanoids [36,58]. On the other hand, Zomborszki et al. (2019) reported that different drying methods did not affect the content of the bioactive compounds of *R. rosea* [59]. Our results supported the first hypothesis that freeze-drying of plant material before the extraction at low temperatures prevents degradation of bioactive compounds and ensures a high yield of bioactive compounds [58,60,61,62]. Long-term vegetative propagation and high genetic diversity [21] of *R. rosea*, together with the freeze-drying method, may explain the high content of the bioactive compounds observed in the current study.

The production of bioactive compounds in *R. rosea* is also influenced by environmental factors such as geographic origin [37,47,53], cultivated-wild and age [14,46], fertilization [20], plant parts and harvesting time [14,53,54,56,63,64], growth factors such as day length and temperature [57], and plant gender [46,56,65]. Genetic factors strongly influence the production of bioactive compound contents in *R. rosea* [21,27,32,40]. However, *R. rosea* plants used in the present study were collected from various geographical locations in Norway and since 2001 were vegetatively propagated under the same environmental conditions in the Norwegian Institute for Bioeconomy Research (NIBIO) germplasm collection. Thus, the high chemo-diversity observed for different clones in this study can be explained by genetic factors rather than environmental influences. Further studies dealing with transcriptomic and gene expression are required.

## 4. Materials and Methods

### 4.1. Materials and Reagents

Seven clones (M1, M2, M3, M4, M5, M6, and M7) of *R. rosea* (Table 5) were selected from the Norwegian germplasm collection of *Rhodiola rosea* comprising 95 different clones collected in 2001 [26]. *R. rosea* clones were vegetatively propagated for the last 16 years at a field at the Norwegian Institute for Bioeconomy Research (NIBIO). The bioactive compounds (rosavin, salidroside, rosin, tyrosol, and cinnamyl alcohol) of these clones were previously analyzed and quantified [26].

In October 2017, 100–150 g of rhizomes of each *R. rosea* clone was collected, washed, and sliced, and two separate methods were used to dry these rhizomes: (i) the samples were dried for 14 h at 70 °C and then stored at room temperature; (ii) the samples were immediately frozen in liquid nitrogen and then freeze-dried at −130 °C for 14 h in 50 mL tubes and then stored at −20 °C until analysis. For each drying method, the fresh weight, dry weight, and the percentage of loss of water (loss of moisture) were determined gravimetrically according to European Pharmacopoeia recommendations, and the results are presented in Table 1. *R. rosea* rhizomes were freeze-dried using a Labconco freeze-dryer (Millrock Technology Inc. Kingston, NY, USA).

### 4.2. Extraction and HPLC Analysis

About 1.0 g of rhizomes was extracted with 50 mL of 70% ethanol in an ultrasonic bath for 30 min at room temperature. After filtration, plant material was twice extracted using additional amounts (25 mL) of 70% ethanol at the same conditions. Extracts were combined, filtered into a 100 mL volumetric flask, and added to 70% ethanol; 3 mL was taken for HPLC-analysis. HPLC analysis was performed as described previously [26]. The reference standards of salidroside, tyrosol, rosavin, rosin, and cinnamyl alcohol were purchased from Sigma-Aldrich, (St Louis, MO, USA). The limit of quantification (LOQ) for the reference substances was 0.25, 0.25, 0.25, 0.2, and 0.25-μg mL^−1^ for salidroside, tyrosol, rosavin, rosin, and cinnamyl alcohol, respectively. The analyses were performed at the same laboratory in St. Petersburg Institute of Pharmacy, Russia, as previously described [26]. The concentrations of salidroside, tyrosol, rosavin, rosin, and cinnamyl alcohol were calculated for absolute dry mass of rhizomes. All chemical analyses were performed in triplicate for each sample to ensure reproducibility, and the means were compared by Fisher’s least significant difference (LSD) test.

### 4.3. Statistical Analyses

#### 4.3.1. Correlation Analyses

Before analyses, data sets were checked for normality of their distributions. All data were normally distributed and thus were analyzed in their raw form using the PROC MIXED function of SAS (SAS, 2002–2003) and including Tukey’s adjustment for multiple comparisons. Stated differences between observations were declared when *p* < 0.05. Pearson product moment correlations between the concentration of each bioactive compound and the concentrations of the other four bioactive compounds were calculated by the PROC CORR function of SAS (SAS, 2002–2003).

#### 4.3.2. Association Analysis

Association between AFLP markers [21] and the production of bioactive compounds of *R. rosea* clones in the present study was estimated through stepwise multiple regression analysis, where each chemical compound was treated as a dependent variable while the AFLP marker was treated as an independent variable as described by Virk et al. (1996) [66]. To select independent variables for the regression equation, F-values with 0.045 and 0.099 probability were used to enter and remove, respectively, as suggested by Roy and Bargmann (1957) [67] and Affifi and Clark (1990) [68]. The analysis was performed using the MINITAB program. Only results obtained using the freeze-drying method were compared to our previous study [26].

## 5. Conclusions

To the best of our knowledge, this is the first study in which the production of the major phenyletanes and phenylpropanoids rosavin, salidroside, rosin, cinnamyl alcohol, and tyrosol by rhizomes of seven clones of *R. rosea* vegetatively propagated for 16 years is reported. We assume that the high chemo-diversity observed for different clones in this study can be explained by genetic factors rather than environmental influences. Further studies dealing with transcriptomic and gene expression are required to confirm this hypothesis. The freeze-drying method increased the concentration of all phenyletanes and phenylpropanoids in rhizomes compared with conventional drying at 70 °C. Long-term vegetative propagation and high genetic diversity of *R. rosea*, together with the freeze-drying method, may have led to the high content of the bioactive compounds observed in the current study.

## Figures and Tables

**Table 1:** The amount of water lost using freeze-drying at −130 °C and drying at 70 °C in seven *R. rosea* clones cultivated in the germplasm collection in Norway since 2001 (mean ± standard deviation).

Clone Id			Freeze-Drying at −130 °C		Drying at 70 °C	
	Fresh-Weight (g)	Dry Weight (g)	% of Water Loss	Fresh-Weight (g)	Dry Weight (g)	% of Water Loss
M1	22.77 ± 1.42	5.21 ± 0.31	77.12	24.96 ± 1.72	5.88 ± 0.64	76.44
M2	23.61 ± 1.61	5.37 ± 0.28	77.26	25.83 ± 1.84	6.08 ± 0.92	76.46
M3	26.39 ± 1.84	6.31 ± 0.42	76.09	25.45 ± 1.81	6.22 ± 0.81	75.56
M4	26.51 ± 1.71	6.12 ± 0.38	76.91	24.68 ± 1.63	5.98 ± 0.73	75.77
M5	27.18 ± 2.02	6.53 ± 0.61	75.97	26.06 ± 2.05	6.44 ± 1.02	75.29
M6	26.24 ± 1.48	5.61 ± 0.84	78.62	27.76 ± 2.14	6.03 ± 1.11	78.28
M7	24.04 ± 1.59	5.97 ± 0.76	75.17	26.54 ± 1.97	6.85 ± 0.94	74.19

**Table 2:** The concentration of rosavin, salidroside, rosin, cinnamyl alcohol, and tyrosol in *R. rosea* rhizomes (collected in 2017) after freeze-drying at −130 °C and drying at 70 °C (mean in mg g^−1^ ± standard deviation).

Compound	Freeze-Drying at −130 °C		Drying at 70 °C	
	Minimum Level	Maximum Level	Minimum Level	Maximum Level
Rosavin	24.40 ± 0.01 *	73.12 ± 1.24 *	17.71 ± 0.91	54.0 ± 3.31
Salidroside	21.92 ± 0.06 **	50.96 ± 0.58 **	12.91 ± 0.81	38.72 ± 1.82
Rosin	0.52 ± 0.02 *	5.84 ± 0.27	0.17 ± 0.03	4.61 ± 0.35
Cinnamyl alcohol	0.43 ± 0.01 *	4.83 ± 0.35 **	0.12 ± 0.01	1.62 ± 0.24
Tyrosol	0.58 ± 0.01 ***	4.80 ± 0.43 *	0.23 ± 0.03	1.64 ± 0.37

* *p* < 0.05, ** *p* < 0.01, and *** *p* < 0.001 (freeze-drying at −130 °C vs. drying at 70 °C).

**Table 3:** Pearson correlation coefficients (r) for the correlation analysis between the concentration of bioactive compounds produced by *R. rosea ^#^*.

	Rosavin	Salidroside	Rosin	Cinnamyl Alcohol	Tyrosol
**Rosavin**	1.0000				
**Salidroside**	0.9025 ***	1.00000			
**Rosin**	0.6431 **	0.2648	1.0000		
**Cinnamyl alcohol**	0.4863 *	0.5614 *	0.7235 **	1.0000	
**Tyrosol**	0.6274 **	0.6349 *	0.6184 **	0.4356 *	1.0000

^#^ Results obtained using the freeze-drying method are presented in the table. * *p* < 0.05, ** *p* < 0.01, and *** *p* < 0.001.

**Table 4:** Comparison of the content of rosavin, salidroside, rosin, cinnamyl alcohol, and tyrosol in *R. rosea* reported in this study and in the literature. Values given are in mg g^−1^.

Origin of Plants	Rosavin	Salidroside	Rosin	Cinnamyl Alcohol	Tyrosol	Literature Reference
Norway	73.120	50.910	5.831	4.820	4.837	This study ^1,D^
Norway	85.950	12.850	4.750	1.180	2.150	[26] ^D^
China	ns	11.100	ns	ns	2.200	[44] ^D^
China	0.650	11.140	3.580	ns	1.120	[27] ^ND^
Finland	0.790	0.280	0.120	0.080	ns	[45] ^D^
Finland	18.140	7.380	ns	ns	ns	[46] ^D^
Lithuania	3.688	1.352	1.603	ns	ns	[47] ^D^
Mongolia	18.700	13.100	ns	18.900	ns	[37] ^D^
Poland	27.900	4.000	ns	10.500	ns	[37] ^D^
Russia	25.000	12.000	ns	ns	ns	[48] ^D^
Russia	ns	ns	1.000	ns	ns	[49] ^ND^
Russia	4.110	0.930	0.530	0.300	ns	[45] ^D^
Russia	0.562	1.624	2.574	ns	ns	[47] ^D^
Sweden	50.700	0.000	ns	15.600	ns	[37] ^D^
China	0.027	0.271	0.180	ns	0.040	[50] ^ND^
Pollen	3.61	6.790	ns	ns	1.890	[35] ^D^
Germany	3.67	3.08	0.70	1.06	0.460	[36] ^ND^
Bulgaria	19.7	26.700	0.412	ns	ns	[23] ^ND^
USA	3.500	2.700	0.800	ns	ns	[28] ^ND^
Norway	18.10	17.70	2.00	ns	1.60	[51] ^ND^
Bulgaria	ns	14.6	ns	ns	ns	[19] ^D^
Canada	21.40	17.61	3.11	ns	2.82	[52] ^D^
UK	4.20	1.200	ns	ns	ns	[53] ^D^
Austria	2.70	27.30	1.50	8.80	18.4	[54] ^D^
Norway	3.63	21.19	ns	0.20	0.41	[34] ^D^
Poland	9.770	1.970	4.624	ns	0.381	[14] ^D^

^1^ Results obtained using the freeze-drying method are presented in the table. ^D^ = concentrations were calculated for absolute dry mass of rhizomes; ^ND^ = not described (mostly commercially available products); ns = not studied.

**Table 5:** The seven clones of *Rhodiola rosea* from the Norwegian germplasm collection. For each clone, information about the regional (SW = south-west, ME = mid-east, *N* = north) and county origin, gender (M = male, F = female), and latitude and longitude is presented.

Clone Id	Region	County	Gender	Latitude	Longitude
M1	SW	Rogland	M	59°39′ N	06 18′ E
M2	N	Nordland	M	68°05′ N	15 38′ E
M3	SW	Sogn og Fjordane	M	61°10′ N	06 01′ E
M4	N	Finnmark	M	70°36′ N	27 00′ E
M5	ME	Sør-Trondelag	M	63°09′ N	11 39′ E
M6	SW	Møre og Romsdal	M	62°25′ N	07 59′ E
M7	N	Finnmark	F	70°37′ N	27 00′ E
